# Alterations in gut microbiome composition, function, and rhythmicity are modulated by time‐restricted feeding in Alzheimer’s disease mice accompanying rescue of brain pathology and memory

**DOI:** 10.1002/alz.090015

**Published:** 2025-01-03

**Authors:** Daniel S. Whittaker, Erica S Maissy, R. Alexander Richter, Simone Zuffa, Stephany Z Flores Ramos, Amir Zarrinpar, Paula Desplats

**Affiliations:** ^1^ University of California, San Diego, La Jolla, CA USA

## Abstract

**Background:**

Studies using Alzheimer’s disease (AD) models suggest that gut bacteria contribute to amyloid pathology and systemic inflammation. Further, gut‐derived metabolites serve critical roles in regulating cholesterol, blood‐brain barrier permeability, neuroinflammation, and circadian rhythms. Recent studies from the Alzheimer’s Disease Neuroimaging Initiative have shown that serum‐based gut‐derived metabolites are associated with AD biomarkers and cognitive impairment. We recently reported a time‐restricted feeding (TRF) intervention that restored brain transcription, increased Aβ clearance, reduced amyloid deposition, and improved memory deficits in AD mice (PMID:37607543). Here we investigated gut microbiome alterations in the APP23 mouse stool and terminal ileum and evaluated the role of the microbiome and metabolome in the beneficial effects of TRF.

**Methods:**

Adult male and female APP23 transgenic (TG) and littermate non‐transgenic (NTG) mice (n = 3‐4/sex/genotype/condition) underwent ad libitum feeding (ALF) or a TRF protocol consisting of 6‐hours of active‐phase feeding followed by 18‐hours fasting for 3‐months. Metabolomics, metagenomics and metatranscriptomics were performed on ileum and stool (collected every 4‐hours for 24‐hours) from mice used in our TRF intervention study.

**Results:**

Metagenomic analyses revealed that the stool microbiome composition and genomic functions were altered in APP23 TG compared to NTG mice and were further uniquely modulated in TG mice on TRF. Notably, stool metabolites relating to metabolism and neuroimmune function were differentially abundant in TG mice and partially restored by TRF. The stool microbiome and metabolome presented distinct diurnal cycling dynamics. TG mice showed a significant loss of rhythmic genomes which were markedly increased by TRF, indicative of broad entrainment of microbial rhythmicity. Ileal diurnal dynamics further differentiated TG mice on TRF. Ileal metatranscriptomic analysis revealed that TRF also reversed bacterial compositional and functional alterations in TG mice, including the attenuation of elevated inflammation‐related bacterial transcripts.

**Conclusions:**

TG mice showed alterations in microbiome composition, function, and rhythmicity. The unique microbiome induced by TRF regulated functions and metabolites implicated in AD and may represent one of the pathways by which TRF rescued pathology and cognition in AD mice.

